# Ion channel Piezo1 activation aggravates the endothelial dysfunction under a high glucose environment

**DOI:** 10.1186/s12933-024-02238-7

**Published:** 2024-05-03

**Authors:** Xiaoyu Zhang, Shaoqiu Leng, Xinyue Liu, Xiang Hu, Yan Liu, Xin Li, Qi Feng, Wei Guo, Nailin Li, Zi Sheng, Shuwen Wang, Jun Peng

**Affiliations:** 1 Department of Hematology, Qilu Hospital, Cheeloo College of Medicine, Shandong University, Jinan, China; 2 State Key Laboratory of Experimental Hematology, National Clinical Research Center for Blood Diseases, Institute of Hematology and Blood Diseases Hospital, Chinese Academy of Medical Sciences and Peking Union Medical College, Tianjin, China; 3 Advanced Medical Research Institute, Shandong University, Jinan, China; 4 Shandong Key Laboratory of Immunochematology, Qilu Hospital, Cheeloo College of Medicine, Shandong University, Jinan, China; 5 National Key Laboratory for Innovation and Transformation of Luobing Theory; the Key Laboratory of Cardiovascular Remodeling and Function Research, Chinese Ministry of Education, Chinese National Health Commission and Chinese Academy of Medical Sciences, Qilu Hospital, Cheeloo College of Medicine, Shandong University, Jinan, China; 6 Institute of Hematology, the First Affiliated Hospital of Zhejiang University School of Medicine, Zhejiang University, Hangzhou, 310029 China; 7 Department of Medicine-Solna, Cardiovascular Medicine Unit, Karolinska Institutet, Stockholm, Sweden

**Keywords:** Piezo1, Endothelial dysfunction, Hyperglycemia, Oxidative stress, Nrf2

## Abstract

**Background:**

Vasculopathy is the most common complication of diabetes. Endothelial cells located in the innermost layer of blood vessels are constantly affected by blood flow or vascular components; thus, their mechanosensitivity plays an important role in mediating vascular regulation. Endothelial damage, one of the main causes of hyperglycemic vascular complications, has been extensively studied. However, the role of mechanosensitive signaling in hyperglycemic endothelial damage remains unclear.

**Methods:**

Vascular endothelial-specific Piezo1 knockout mice were generated to investigate the effects of Piezo1 on Streptozotocin-induced hyperglycemia and vascular endothelial injury. In vitro activation or knockdown of Piezo1 was performed to evaluate the effects on the proliferation, migration, and tubular function of human umbilical vein endothelial cells in high glucose. Reactive oxygen species production, mitochondrial membrane potential alternations, and oxidative stress-related products were used to assess the extent of oxidative stress damage caused by Piezo1 activation.

**Results:**

Our study found that in VE^CreERT2^;Piezo1^flox/flox^ mice with Piezo1 conditional knockout in vascular endothelial cells, Piezo1 deficiency alleviated streptozotocin-induced hyperglycemia with reduced apoptosis and abscission of thoracic aortic endothelial cells, and decreased the inflammatory response of aortic tissue caused by high glucose. Moreover, the knockout of Piezo1 showed a thinner thoracic aortic wall, reduced tunica media damage, and increased endothelial nitric oxide synthase expression in transgenic mice, indicating the relief of endothelial damage caused by hyperglycemia. We also showed that Piezo1 activation aggravated oxidative stress injury and resulted in severe dysfunction through the Ca^2+^-induced CaMKII-Nrf2 axis in human umbilical vein endothelial cells. In Piezo1 conditional knockout mice, Piezo1 deficiency partially restored superoxide dismutase activity and reduced malondialdehyde content in the thoracic aorta. Mechanistically, Piezo1 deficiency decreased CaMKII phosphorylation and restored the expression of Nrf2 and its downstream molecules HO-1 and NQO1.

**Conclusion:**

In summary, our study revealed that Piezo1 is involved in high glucose-induced oxidative stress injury and aggravated endothelial dysfunction, which have great significance for alleviating endothelial damage caused by hyperglycemia.

**Supplementary Information:**

The online version contains supplementary material available at 10.1186/s12933-024-02238-7.

## Introduction

Diabetes is a chronic metabolic disease characterized by elevated circulating glucose levels, which is accompanied by diabetic vasculopathy and abnormal blood flow in the later stages [1, 2]. High glucose leads to endothelial dysfunction, which is the earliest and most fundamental pathological alteration in diabetes [3–5].

High glucose-induced endothelial metabolic abnormalities result in excessive production of reactive oxygen species (ROS), thereby leading to oxidative stress injury and inflammatory responses during vascular remodeling [6]. Meanwhile, the pro-oxidative environment in diabetes may destroy endothelial function by reducing nitric oxide (NO) synthesis and attenuate essential anti-atherogenic and euglycemic vascular effects [7], resulting in a vicious cycle of diabetic adverse vascular events. Several strategies have been developed to improve endothelial function, and the indispensable role of antioxidants in restoring endothelium-dependent vascular function is highlighted through various evidence [8].

Blood flow creates mechanical stress, which has a significant impact on vascular function. Studies have shown that hemodynamic changes are related to atherothrombosis [9] and the progression of atherosclerotic plaque [10], thereby contributing to the transformation of stable to unstable plaque [11]. The viscoelastic and thixotropic behaviors of blood thixotropies are significantly increased in diabetes [1]. Endothelial cells distributed in the innermost layer of blood vessels can feel mechanical stresses, including unidirectional laminar flow and oscillatory shear, and produce NO and ROS [12], which in turn affect endothelial function or mediate contractility changes and vascular remodeling through inflammation, oxidative stress, endoplasmic reticulum stress, autophagy, endothelial-mesenchymal transition, epigenetic regulation and endothelial metabolic adaptation [13–15].

Piezo1 is an important non-selective mechanosensitive ion channel that can be activated by pressure, flow-related shear stress, cyclical hydrostatic pressure, and the chemical agonist Yoda1 [16–18]. Piezo1 activation-induced Ca^2+^ influx has been shown to play important roles in erythrocyte homeostasis, myocardial contraction, macrophage inflammatory responses, and bone remodeling [19–22]. Endothelial Piezo1 is involved in embryonic angiogenesis and vasodilation in adults [23–25]. Studies have revealed the mechanism by which diabetic vasculopathy and abnormal blood flow activate Piezo1 to regulate thrombosis; however, whether it is also related to endothelial cell dysfunction remains to be studied [26].

This study aimed to explore the role of endothelial Piezo1 in a high glucose environment by generating VE^CreERT2^;Piezo1^flox/flox^ mice with Piezo1 conditional knockout in endothelial cells. Surprisingly, we found that streptozotocin (STZ)-induced hyperglycemia was alleviated in Piezo1-deficient mice after 6 months. Additionally, Piezo1 deficiency reduced the apoptosis of thoracic aortic endothelial cells and alleviated the inflammatory response in the aortic tissue caused by high glucose. Our study revealed that Piezo1 activation aggravated oxidative stress injury and resulted in severe dysfunction by activation of the Ca^2+^-calcium/calmodulin-dependent protein kinase II (CaMKII) pathway and inhibition of Nrf2 with its downstream molecules in human umbilical vein endothelial cells (HUVECs). Conditionally, Piezo1-deficient mice were also confirmed to reduce CaMKII phosphorylation and restore the expression of Nrf2 and its downstream molecules.

## Methods

### Animals

To generate tamoxifen (TAM)-induced disruption of Piezo1 in the endothelium, Piezo1^flox/flox^ mice were crossed with VE-Cadherin^creERT2^ mice and inbred to obtain vascular endothelial-specific Piezo1 conditional knockout mice VE^CreERT2^;Piezo1^flox/flox^, as reported elsewhere [27–30]. VE^CreERT2^;Piezo1^flox/flox^ and Piezo1^flox/flox^ mice were administered with 80 mg/kg/day STZ (Sigma-Aldrich, USA) by intraperitoneal injection after overnight fasting for three consecutive days to establish a hyperglycemia model. One week later, the model was considered successful when the fasting blood glucose level was greater than 7.9 mmol/L with symptoms of polydipsia, polyuria, and polyphagia. VE^CreERT2^;Piezo1^flox/flox^ mice and their control littermates were maintained in a specific pathogen-free (SPF) facility with 23 °C and 40–60% humidity, 12 h light/ dark cycle. All animals were euthanized using chloral hydrate. All animal experiments followed the Guidelines for Animal Protection and Use of the People’s Republic of China and carried out with the approval of Animal Ethics Committee of the Cheeloo College of Medicine, Shandong University.

### Mouse endothelial cells isolation

Mice used for sampling were sacrificed under anesthesia and then dissected to expose the chest and abdomen. Following isolation, the kidneys and lungs were minced and digested with 0.1% collagenase I at 37 °C for 45 min. Then, the organs within the chest cavity were gently pushed aside, the thoracic aorta was bluntly dissected from the spine and carefully cleared of extraneous fat and connective tissue. The isolated thoracic aorta was inverted on the inner surface using dissecting forceps under a dissecting microscope, followed by digestion with 0.1% collagenase I at 37 °C for 30 min, with periodic agitation and thorough shaking. The digested cells were sorted for endothelial cells using anti-CD31 magnetic beads separation kit (Miltenyi Biotec, Germany) after lysis of red blood cells. The purity of the isolated cells was found to be > 90% by flow cytometry.

### Cell culture

HUVECs cell line were purchased from the ZhongQiaoXinZhou Biotechnology Company (Shanghai, China), and supplemented in 1640 medium with 10% fetal bovine serum (FBS) and 1% penicillin/streptomycin in an incubator containing 95% air and 5% carbon dioxide (CO_2_) at 37 °C until the start of the experiment. HUVECs were treated with 1640 medium consisting of either normal glucose (NG, 5.5 mM) (Sigma-Aldrich, USA) or high glucose (HG, 30 mM), D-mannitol (NG + MAN, 30 mM:5.5 mM glucose + 24.5 mM D-mannitol) served as the osmotic control for HG. KN93 (10 μM) and ML385 (10 mM) (MedChemExpress, USA) were pretreated for half an hour before Yoda1 (2 μM) (Selleck Chemicals, USA) administration for signaling analysis. Lipofectamine 3000 transfection reagent (Invitrogen, USA) was used according to the manufacturer’s instructions for siRNA transfection. The siRNA sequences are listed in Additional file 3: Table S1.

### Cell proliferation and apoptosis

CCK8 assay was used to detect cell proliferation. The treated HUVECs were added with 10c CCK8 solution (Bestbio, China) per well and incubated at 37 °C for 4 h, followed by measurement of the absorbance at 450 nm with an enzyme label for five consecutive days. An apoptosis detection kit (Bestbio, China) was used to detect apoptosis in treated HUVECs using flow cytometry according to the manufacturer’s instructions.

### Transwell assay

A total of 4 × 10^4^ HUVECs suspended in a medium without serum were added to the Transwell chamber (Corning Life Sciences, USA), which was then inserted into the wall containing the same medium with 10% FBS. After incubation for 16 h, the cells in the upper chamber were gently removed. The cells on the Transwell membrane were fixed with methanol and stained with hematoxylin. The number of migrated cells was counted in three random fields captured using a microscope.

### Wound healing assay

A total of 5 × 10^5^ HUVECs were seeded in each well of six-well plates and incubated overnight. After a straight scratch wound was generated, the floating cells were removed and gently washed three times with phosphate buffered saline (PBS). The cells were incubated in 1% FBS medium and images were captured at the same position at 0, 12, and 24 h. The cell migration activity was calculated by the area that healed at different time point relative to 0 h.

### In vitro angiogenesis assay

A total of 3 × 10^4^ HUVECs were plated in 48-well plates precoated with 50 μL/well growth factor–reduced Matrigel (Corning Life Sciences, USA) and incubated at 37 °C for 16 h. The formation of lumen-like structures was observed under a microscope and the number of vascular cavities was counted in three random fields.

### ROS detection

A total of 5 × 10^5^ HUVECs were seeded in each well of six-well plates and incubated overnight; 1–2 ml serum-free medium diluted with 2,7-dichlorodi-hydro fluorescein diacetate (DCFH-DA) solution (Yeasen, China) was added with a working concentration of 0.5 μM, incubated at 37 °C for 5 min in the dark, then washed with buffer solution. The fluorescence was visualized by fluorescence microscopy, and then the cells were digested to measure the mean fluorescence intensity by flow cytometry.

### MitoSOX assay

Mitochondrial superoxide production was detected using the MitoSOX assay. The pretreated HUVECs were placed in six-well plates; 1–2 ml serum-free medium diluted with MitoSOX solution (Yeasen, China) was added with a working concentration of 1 μM, incubated at 37 °C for 15 min in the dark, washed with buffer solution, followed by observation and photography under a fluorescence microscope.

### JC-1 staining

The pretreated HUVECs were seeded in six-well plates and added into JC-1 staining solution (5 μg/mL) (Beyotime, China) of the same volume as the medium, incubated at 37 °C for 20 min in the dark, and washed with PBS. Relative amounts of mitochondrial JC-1 monomers or aggregates were measured by fluorescence microscopy using 488 nm and 561 nm lasers. The ratio of red/green fluorescence intensity of JC-1 was normalized to access the loss of mitochondrial membrane potential.

### Oxidative stress-level assays

The levels of MDA and SOD activity in HUVECs and mouse aortic tissue were quantitatively measured using MDA and SOD assay kits (Nanjing Jiancheng Bioengineering Institute, China), respectively. The treated HUVECs were ultrasonically broken, and the corresponding detection solutions were added according to the manufacturer’s instructions. After cultivation with or without a 95 °C-water bath, the absorbance at OD530 and OD450 was detected respectively to quantitatively determine the intracellular MDA and SOD content. Intracellular superoxide levels were quantitatively measured using a superoxide assay kit (Beyotime, China). The culture medium of the pretreated HUVECs was collected and added to the assay kit according to the manufacturer’s instructions. After cultivation for 3 min at 37 °C, OD450 and OD600 were measured to quantitatively determine the superoxide.

### Bioinformatics analysis

The RNA-seq data (GSE54387) and snRNA-seq datasets (GSE131882) were downloaded from the Gene Expression Omnibus (GEO) database (https://www.ncbi.nlm.nih.gov/geo/) to study the differential expression of Piezo1 between the healthy and diet-induced obesity or diabetes groups. Differentially expressed genes (DEGs) were defined as |logFC| > 0.5 and p value < 0.05. R software v.2.2.2 (https://www.r-project.org/) and Seurat v4.3.0 package (https://satijalab.org/seurat/) were used for the analysis.

### HE staining of aortic ring sections

Four weeks or six months after the onset of STZ-induced diabetes, the mice were sacrificed under anesthesia. The thoracic aorta was dissected, surrounding connective tissue was removed, and frozen sections with a thickness of 5 μm were prepared after fixation with 4% paraformaldehyde. Sections were stained with hematoxylin for 15 min in a tray containing 0.1% eosin for 5 min after fixation and membrane penetration, dehydrated using a gradient of absolute ethanol and xylene, and finally sealed using neutral gum. The stained sections were observed at microscope and photographed.

### TUNEL assay

Fixed frozen sections of the mouse thoracic aorta were stained using an in-situ cell Death Detection kit (Roche Applied Science, Germany) and then examined by fluorescence microscopy using a 488 nm laser.

### RNA extraction and RT-PCR

An RNA extraction kit (Yishan Biotechnology Co., China) was used to lyse and extract RNA from the HUVECs, mouse endothelial cells and mouse thoracic aortic tissues. The mRNA was then converted into complementary DNA (cDNA) using the Prime Script RT reagent kit (Takara Bio Inc., Japan) according to the recommended protocol. The data were measured using a Light Cycler 480 System (Roche Applied Science, Germany). The allele-specific primers are listed in Additional file 3: Table S1.

### Calcium imaging

HUVECs were cultured with the indicated conditions. Thereafter, the cells were loaded with Fluo-4 AM (2.5 μM) (TermoFisher, USA) for 15–30 min in PBS at 37 °C to observe calcium influx. The intracellular calcium ions were examined by fluorescence microscopy using a 488 nm laser.

### Western blot

Treated HUVECs were digested with trypsin and collected. Mouse endothelial cells were separated by magnetic beads and centrifuged. Mouse aortic tissues were frozen in liquid nitrogen and ground after harvesting. Total protein was extracted using a protein extraction kit (BestBio Company, China) following the manufacturer’s instructions. Protein concentration was measured using a bicinchoninic acid (BCA) protein assay kit (Beyotime Biotechnology, China). Denture proteins were separated by 10% sodium dodecyl-sulfate polyacrylamide gel electrophoresis (SDS-PAGE) (Life Technologies, USA), transferred to a poly vinylidene fluoride (PVDF) membrane (Millipore Sigma, USA) and blocked with 5% skimmed milk at RT for 1 h. The membranes were then incubated with primary antibodies at 4 °C overnight on a shaker, followed by wash with 1 × Tris-buffered saline containing Tween (TBST). Then membranes were incubated with HRP-linked secondary antibodies at RT for 1 h. The protein bands were visualized by an ECL kit (Beyotime, China) on a SageCapture Chemiluminescent imaging system (ChampChemi 610 Plus, China). The intensity (area X density) of the individual band on Western blots was quantified and calculated by ImageJ software. β-actin served as a loading control. Antibodies against CaMKII (A0198), Nrf2(A0674), HO-1(A19062), NQO1(A22290), eNOS (A3774), and secondary antibodies HRP-conjugated β-actin monoclonal (HRP-60008) were purchased from Abclonal (Guangzhou, China). Phospho-CaMKII (Thr286) was purchased from Cell Signaling Technology (Danvers, USA). All antibodies were dissolved and preserved according to the manufacturer’s instructions.

### Statistical analysis

All experiments were repeated at least three times. Data are presented as mean ± standard deviation. The Shapiro–Wilk test was used for normality tests. Comparisons between groups were performed using one-way analysis of variance (ANOVA), two-way ANOVA, or unpaired two-tailed Student’s t tests. Statistical significance was set at P < 0.05.

## Results

### Conditionally endothelial Piezo1-deficient mice alleviated STZ-induced hyperglycemia

**Fig. 1:**
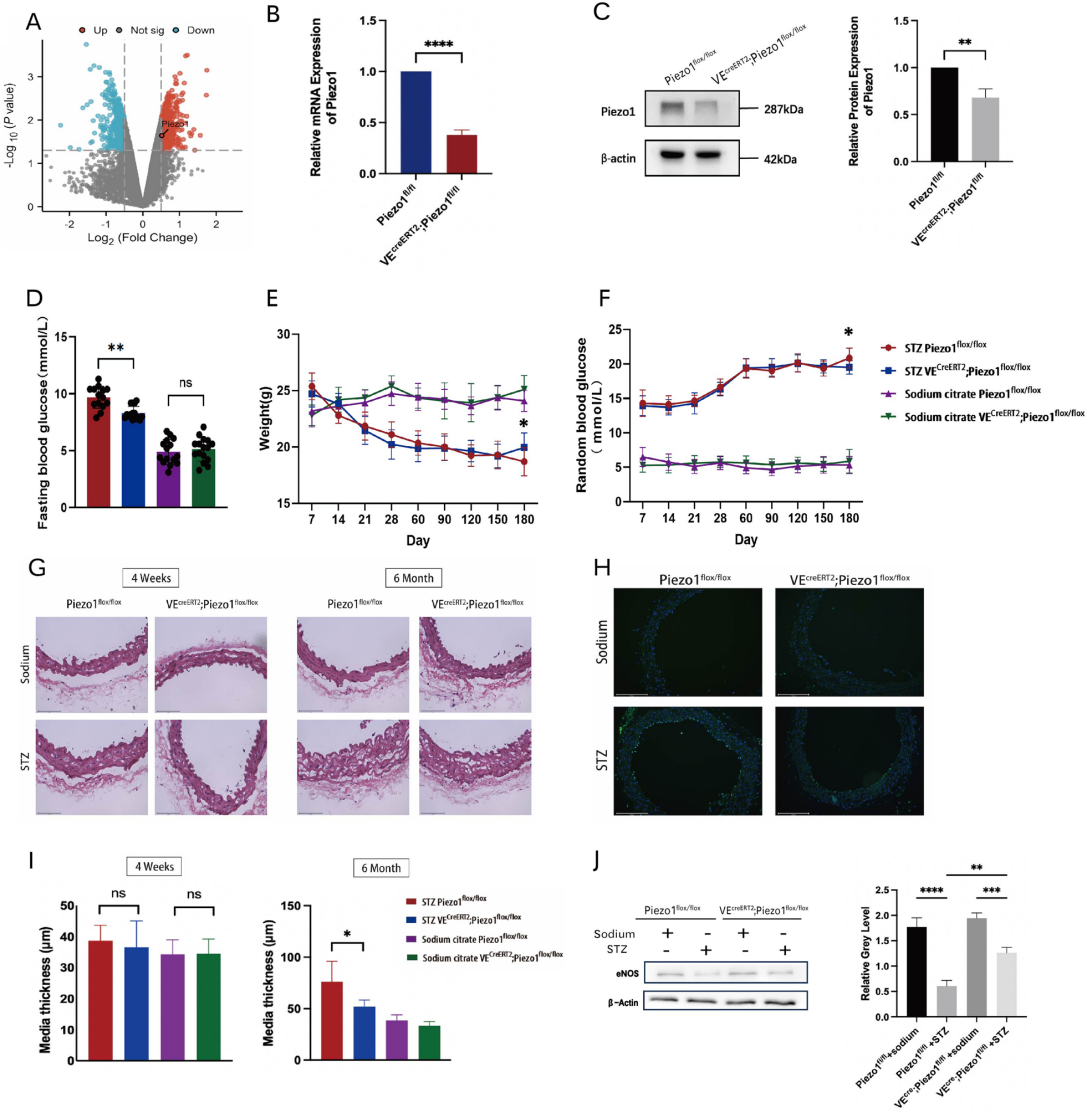
Conditionally endothelial Piezo1-deficient mice alleviated STZ-induced long-term hyperglycemia in diabetes. Piezo1^flox/flox^ and VE^creERT2^;Piezo1^flox/flox^ mice were administrated with 80 mg/kg/d STZ or sodium citrate by intraperitoneal injection after overnight fasting for three consecutive days. **A** Volcano map showing Piezo1 expression of induced pluripotent stem cell-derived endothelial cells in diet-induced hyperglycemic obesity mice compared with healthy mice. **B** The mRNA level of Piezo1 in thoracic aortic endothelial cells of Piezo1^flox/flox^ and VE^creERT2^;Piezo1^flox/flox^ mice relative to GAPDH (n = 3). **C** The protein expression of Piezo1 in thoracic aortic endothelial cells of Piezo1^flox/flox^ and VE^creERT2^;Piezo1^flox/flox^ mice relative to β-actin (n = 3). **D** Fasting blood glucose after a 16-h fast of six-month hyperglycemic mice (n = 14 to 16). **E** Body weight changes after intraperitoneal injection of STZ (n = 11). **F** Dynamic changes in random blood glucose after intraperitoneal injection of STZ (n = 11). **G** Representative images of HE-stained thoracic aorta sections isolated from four-week hyperglycemic mice and 6-month hyperglycemic mice. **H** Representative confocal images of TUNEL-stained thoracic aorta sections isolated from four-week hyperglycemic mice. **I** Media thickness of four-week or six-month hyperglycemic mice (n = 3). **J** Expression of e-NOS of Piezo1^flox/flox^ and VE^creERT2^;Piezo1^flox/flox^ mice relative to β-actin analyzed by western blot (n = 3). Statistical significances were calculated using (**B**, **C**) two tail student’s t test, (**D**–**F** and **I**–**J**) two-way ANOVA, Tukey’s multiple comparisons tests. Data are expressed as the mean ± SD. *P < 0.05; **P < 0.01; ****P < 0.0001; ***P < 0.001, ns, not significant

To study the function of Piezo1 in endothelial cells in diabetes, we first analyzed the expression of Piezo1 in induced pluripotent stem cell-derived endothelial cells in diet-induced hyperglycemic obese mice and compared it with healthy mice (GSE54387). Principal component analysis (PCA) revealed a significant difference between endothelial cells from healthy and diet-induced hyperglycemic obese mice (Additional file 1: Fig. S1A). Among the DEGs, Piezo1 was upregulated in endothelial cells from hyperglycemic obese mice, indicating a vital function for Piezo1 in a hyperglycemic environment (Fig. 1A).

To investigate the regulation of endothelial function by Piezo1, Piezo1^flox/flox^ mice were crossed with VE-Cadherin^creERT2^ mice to generated vascular endothelial-specific Piezo1 conditional knockout mice VE^CreERT2^;Piezo1^flox/flox^, and sex-matched littermates (Piezo1^flox/flox^) served as controls. The efficiency of Piezo1 knockout was verified separately at the mRNA and protein levels (Fig. 1B, C and Additional file 1: Fig. S1B). Murine hyperglycemia was induced by an intraperitoneal injection of STZ. One week later, the fasting blood glucose level was > 7.9 mmol/L, indicating that the model was successfully established. Next, we observed the glycemic mice over a period of six months. Under normal physiological conditions, differences in body weight or random blood glucose were not observed between VE^CreERT2^;Piezo1^flox/flox^ and Piezo1^flox/flox^ mice, implying that knocking out Piezo1 in the vascular endothelial cells does not affect the normal physiological state. Meanwhile, in hyperglycemic mice, we did not find any difference in body weight and random blood glucose between VE^CreERT2^;Piezo1^flox/flox^ and Piezo1^flox/flox^ mice during the first five months. However, VE^CreERT2^;Piezo1^flox/flox^ mice showed markedly relieved STZ-induced hyperglycemic symptoms, with lower fasting blood glucose levels (Fig. 1D), higher body weight (Fig. 1E), and lower random blood glucose levels (Fig. 1F) in the sixth month after STZ induction. These results suggested that endothelial Piezo1 is activated under hyperglycemic conditions, whereas Piezo1 deficiency in vascular endothelial cells alleviates STZ-induced long-term hyperglycemia. To directly assess the effect of Piezo1 knockdown on endothelial function, thoracic aortas were isolated from Piezo1^flox/flox^ and VE^CreERT2^;Piezo1^flox/flox^ mice. HE staining showed that the thoracic aorta endothelial cells were shed four weeks after STZ injection (Fig. 1G), while TUNEL staining showed that Piezo1^flox/flox^ mice had a more obvious endothelial cell apoptosis compared to VE^CreERT2^;Piezo1^flox/flox^ mice (Fig. 1H), suggesting that Piezo1 deficiency alleviated endothelial cell damage in hyperglycemic mice. After 6 months of hyperglycemia, HE staining showed a larger de-endothelialized region and thickened tunica media in the aortic endothelium of Piezo1^flox/flox^ mice (Fig. 1I), accompanied by vacuolization of the vascular smooth muscle, indicating that Piezo1 activation could aggravate irreversible endothelial damage in prolonged hyperglycemia. Additionally, western blotting showed lower expression of endothelial nitric oxide synthase (eNOS) in Piezo1^flox/flox^ mice (Fig. 1J), which is consistent with the synchronous increase in the blood glucose levels of Piezo1^flox/flox^ mice compared with VE^CreERT2^;Piezo1^flox/flox^ mice. This suggests that the long-term activation of Piezo1 under high glucose environment continues to aggravate vascular endothelial cell damage, while knocking out Piezo1 can partially alleviate the damage.

### Piezo1 activation aggravates the high glucose-induced endothelial cell dysfunction in vitro

**Fig. 2:**
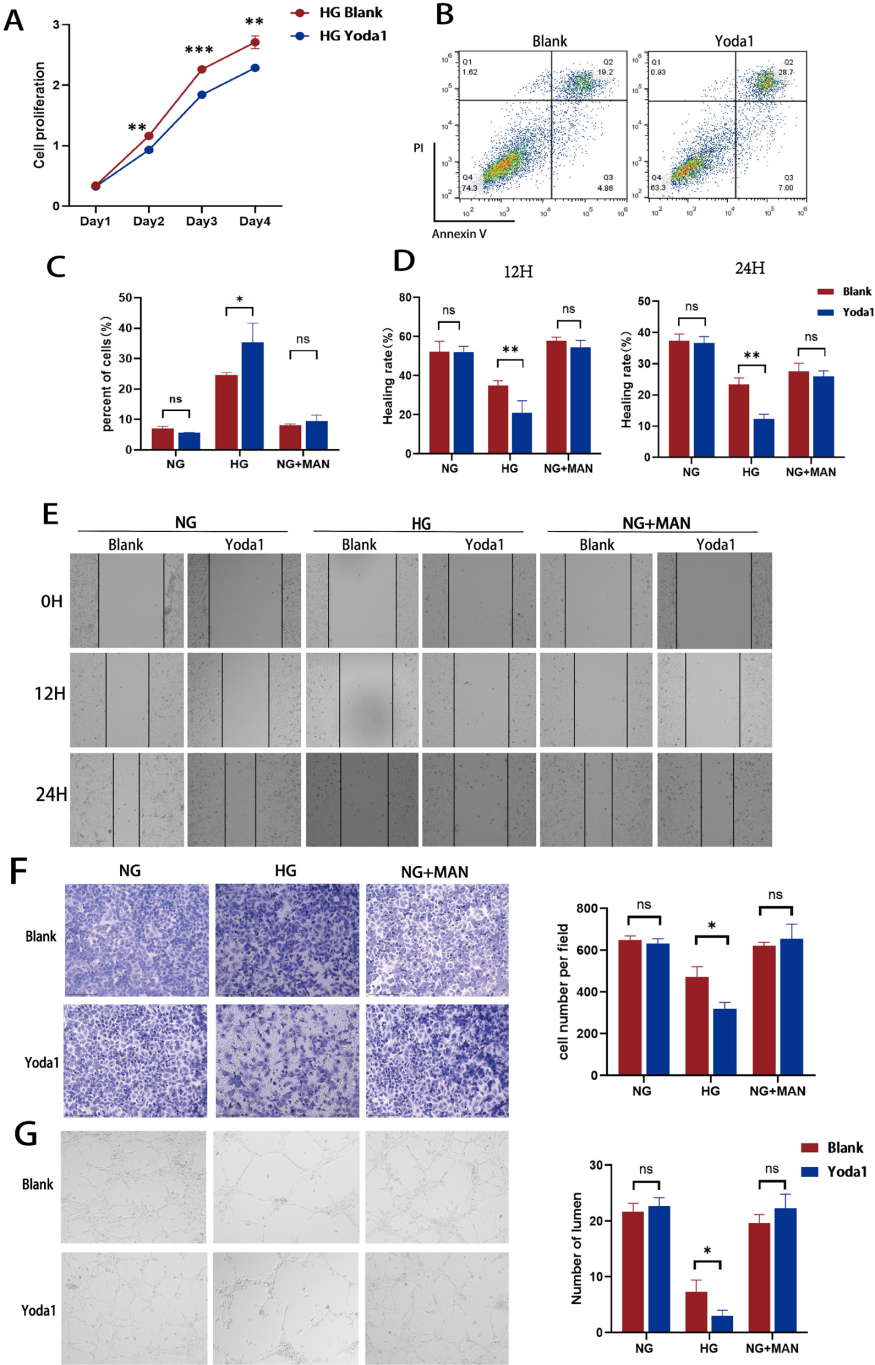
Activation of Piezo1 by Yoda1 aggravated high glucose-induced endothelial dysfunction. HUVECs were cultured in different glucose concentration conditions (NG:5.5 mM glucose, HG:30 mM glucose, NG + MAN:5.5 mM glucose + 24.5 mM mannitol) with or without Yoda1 (2 μM) stimulation. **A** CCK8 assay examined the proliferation of HUVECs in the HG group (n = 3). **B**, **C** The proportion of apoptotic cells after 24 h of culture in the HG group (n = 3). **D** The healing rates of HUVECs at 12 and 24 h of culture (n = 3). **E** Representative images of wound healing assay photographed at 0, 12, and 24 h of culture (100 ×). **F** Representative images of the Transwell assay photographed after hours of culture (200 ×) and the number of cells that passed through the chamber after 16 h of culture (n = 3). **G** Representative images of angiogenesis assay photographed after 20 h of culture (100 ×) and the number of HUVEC-forming lumens at 20 h of culture (n = 3). Statistical significances were calculated using two tail student’s t test. Data are expressed as the mean ± SD. *P < 0.05; **P < 0.01; ***P < 0.001, ns, not significant

**Fig. 3:**
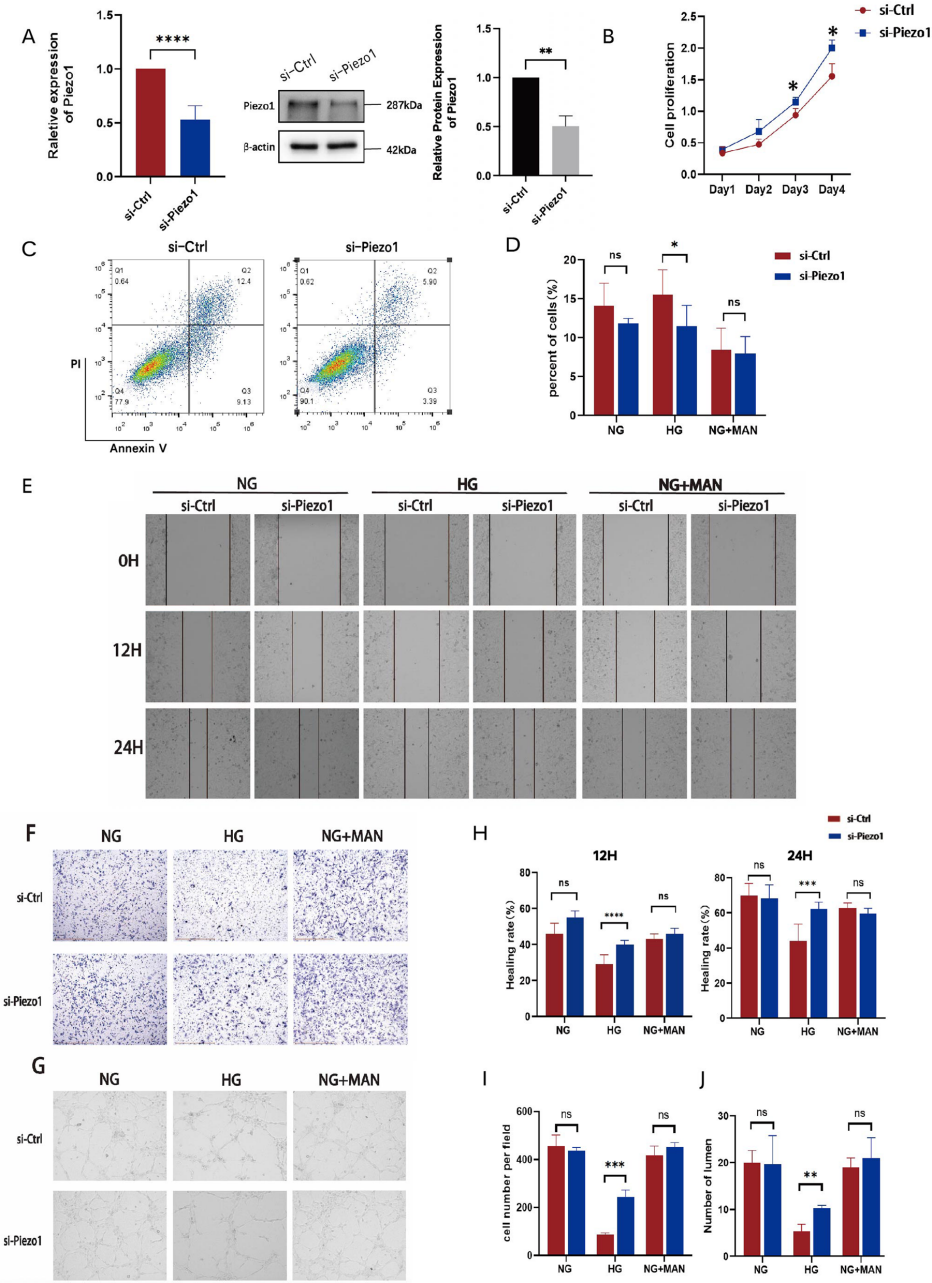
Knockdown of Piezo1 alleviated endothelial cell dysfunction induced by high glucose. si-Ctrl and si-Piezo1 HUVECs were cultured in different glucose concentration conditions (NG:5.5 mM glucose, HG:30 mM glucose, NG + MAN:5.5 mM glucose + 24.5 mM mannitol) with Yoda1 (2 μM) stimulation. **A** The mRNA and protein expression of Piezo1 in si-Ctrl and si-Piezo1 HUVECs relative to GAPDH and β-actin (n = 3). **B** CCK8 assay examined the proliferation of si-Ctrl and si-Piezo1 HUVECs in HG groups (n = 3). **C**, **D** The proportion of apoptotic cells of si-Ctrl and si-Piezo1 HUVECs after 24 h of culture (n = 3). **E** Representative images of wound healing assay photographed at 0, 12, and 24 h of culture (100 ×). **F** Representative images of the Transwell assay photographed after 16 h of culture (100 ×). **G** Representative images of angiogenesis assay photographed after 20 h of culture (100 ×). **H** The healing rates of si-Ctrl and si-Piezo1 HUVECs at 12 and 24 h of culture (n = 3). **I** The number of cells that passed through the chamber after 16 h of culture (n = 3). **J** The number of lumens formed by si-Ctrl and si-Piezo1 HUVECs at 20 h of culture (n = 3). Statistical significances calculated using two tailed student’s t test were used for statistical analysis. Data are expressed as the mean ± SD. *P < 0.05; **P < 0.01; ***P < 0.001; ****P < 0.0001, ns, not significant

To further assess the direct effect of Piezo1 activation on endothelial function in vitro, HUVECs were cultured in different media containing NG (5.5 mM), HG (30 mM), or normal glucose with mannitol (NG + MAN:5.5 mM glucose + 24.5 mM mannitol) alone or with the specific Piezo1 channel agonist Yoda1 (2 μM). Consistent with the endothelial cell damage caused by hyperglycemia, HUVECs treated with HG showed decreased proliferation and increased apoptosis compared to those treated with NG and NG + MAN (Additional file 1: Fig. S1C). However, growth curve analysis and flow cytometry indicated that Yoda1 treatment significantly aggravated cell growth inhibition in the HG group, but did not have a significant effect on the NG and NG + MAN groups (Fig. 2A–C, Additional file 1: Fig. S1C). The results of the Transwell and wound healing assays also showed that HUVECs cultured in media with HG had poor cell migration ability, and Yoda1 significantly restrained the migration of HUVECs compared with the NG and NG + MAN groups (Fig. 2D–F). Meanwhile, the angiogenesis assay indicated that tube formation activity was significantly impaired in HUVECs exposed to HG compared to those maintained in NG and NG + MAN, and Yoda1 significantly increased the functional damage of HUVECs in a HG environment (Fig. 2G). In parallel, we used small interfering RNA (siRNA) to silence Piezo1 in HUVECs cell line, verified the knockdown efficiency at the RNA and protein level (Fig. 3A), and chemically activated Piezo1 with Yoda1. Flow cytometry showed that the knockdown of Piezo1 protected the proliferation of HUVECs treated with HG and reduced the rate of cell apoptosis; however, significant differences were not observed between the NG and NG + MAN-treated groups (Fig. 3B–D, Additional file 1: Fig. S1D-E). In addition, Transwell and wound healing assays were used to detect the effect of Piezo1 on cell migration, which showed that the migration ability of HUVECs increased after the downregulation of Piezo1 expression when incubated with HG, rather than NG or NG + MAN groups (Fig. 3E, F, H, I). Furthermore, the in vitro tube formation assay of si-Piezo1 HUVECs showed a higher tube formation ability in HG medium than that of si-Ctrl HUVECs, indicating that the downregulation of Piezo1 alleviated the damage caused by a HG environment and restored the function of HUVECs (Fig. 3G, J).

### Piezo1 is involved in oxidative stress injury under a HG environment

**Fig. 4:**
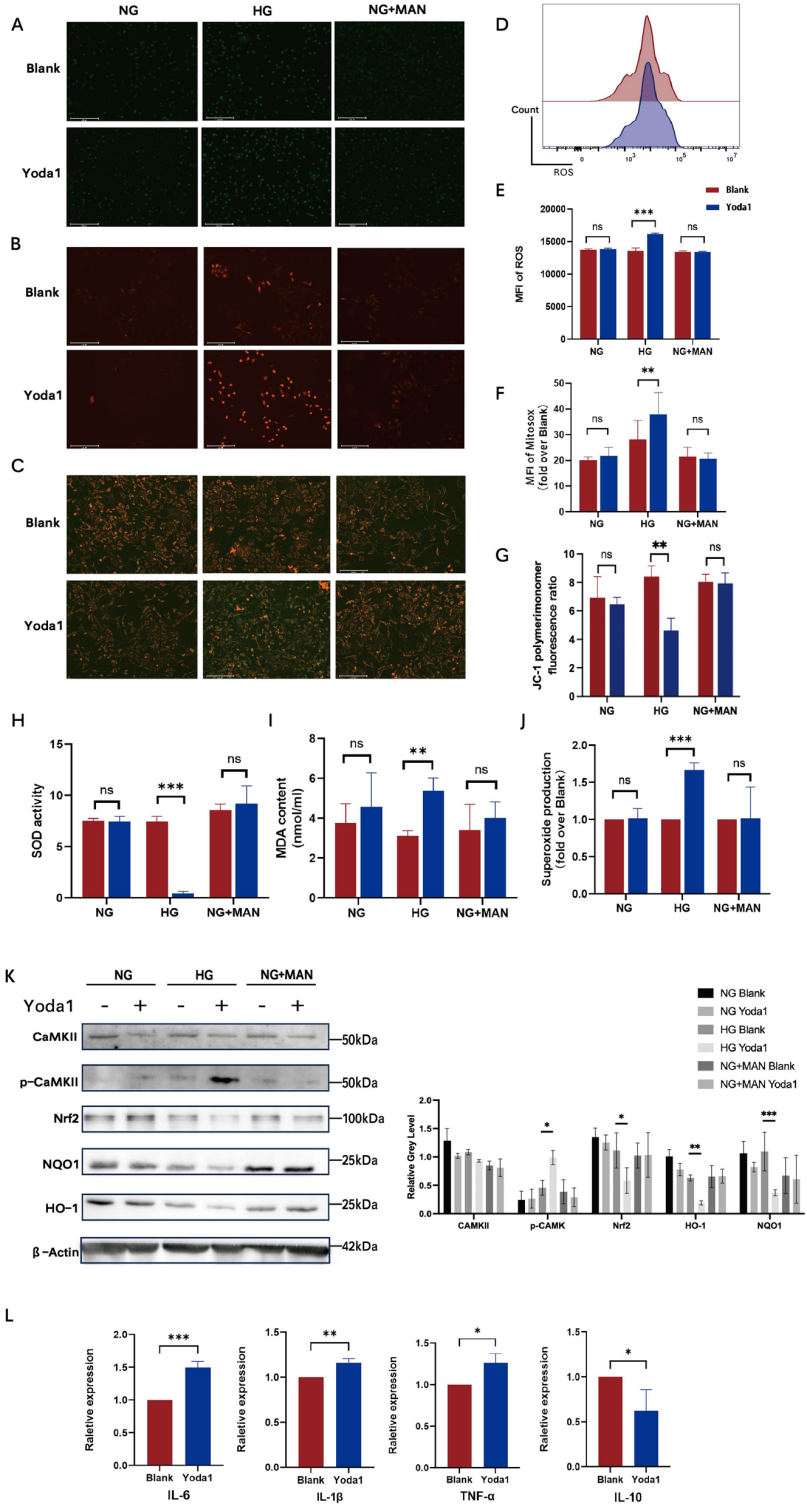
Piezo1 activation aggravated the oxidative stress injury caused by high glucose through Ca2 + /CaMKII and Nrf2/HO-1/NQO1 signaling pathways. HUVECs were cultured in different glucose concentration conditions (NG:5.5 mM glucose, HG:30 mM glucose, NG + MAN:5.5 mM glucose + 24.5 mM mannitol) with or without Yoda1 (2 μM) stimulation. **A** Representative confocal images of ROS production (100 ×). **B** Representative confocal images of MitoSOX stained HUVECs (100 ×). **C** Representative confocal images of mitochondrial membrane potential detected by JC-1 fluorescence staining (100 ×). **D** ROS production measured by flow cytometry in HG groups. **E** ROS production indicated by the mean fluorescence intensity (n = 3). **F** Mitochondrial superoxide production measured by the mitochondria-targeted probe MitoSOX (n = 3). **G** The ratio of polymer monomers to monomers indicated by JC-1 fluorescence. **H** SOD activity of HUVECs measured by ELISA (n = 3). **I** MDA content of HUVECs measured by ELISA (n = 3). **J** Relative superoxide production of HUVECs measured by ELISA (n = 3). **K** Totol-CaMKII, phospho-CaMKII, Nrf2, NQO1, and HO-1 expression of HUVECs analyses by western blot relative to β-actin (n = 3). **L** The mRNA expression of IL-6, TNF-a, IL-1β and IL-10 of HUVECs (n = 3). Statistical significances were calculated using (**E**–**J**) Two tailed student’s t test and (K) two-way ANOVA, Tukey’s multiple comparisons tests. Data are expressed as the mean ± SD. *P < 0.05; **P < 0.01; ***P < 0.001, ns, not significant

**Fig. 5:**
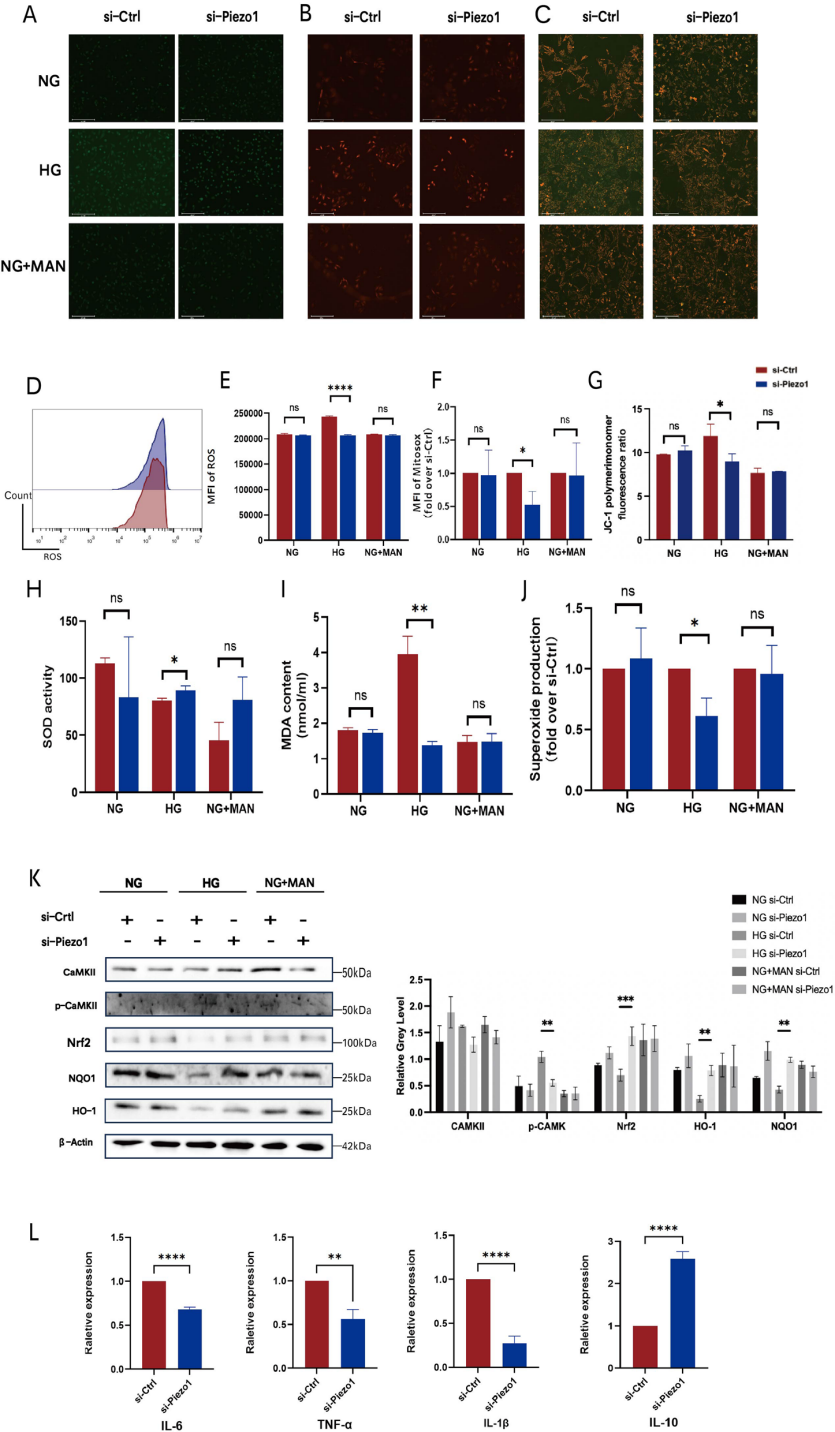
Piezo1 knockdown alleviated the oxidative stress injury caused by high glucose and inhibited the activation of Ca2 + /CaMKII and Nrf2/HO-1/NQO1 signaling pathways. Si-Ctrl and si-Piezo1 HUVECs were cultured in different glucose concentration conditions (NG:5.5 mM glucose; HG:30 mM glucose, NG + MAN:5.5 mM glucose + 24.5 mM mannitol) with Yoda1 stimulation (2 μM). **A** Representative confocal images showing ROS production (100 ×). **B** Representative confocal images of MitoSOX stained si-Ctrl and si-Piezo1 HUVECs (100 ×). **C** Representative confocal images of membrane potential detected by JC-1 fluorescence staining (100 ×). **D** ROS production measured by flow cytometry of si-Ctrl and si-Piezo1 HUVECs (n = 3). **E** ROS production in si-Ctrl and si-Piezo1 HUVECs, as indicated by the mean fluorescence intensity (n = 3). **F** Mitochondrial superoxide production in si-Ctrl and si-Piezo1 HUVECs was measured using the mitochondria-targeting probe MitoSOX (n = 3). **G** The ratio of polymer monomers to monomers indicated by JC-1 fluorescence. (H) SOD activity in si-Ctrl and si-Piezo1 HUVECs was measured by ELISA (n = 3). **I** MDA content of si-Ctrl and si-Piezo1 HUVECs was measured by ELISA (n = 3). **J** Relative superoxide production in si-Ctrl and si-Piezo1 HUVECs as measured by ELISA (n = 3). **K** Totol-CaMKII, phospho-CaMKII, Nrf2, NQO1, and HO-1 expression in si-Ctrl HUVECs and si-Piezo1 HUVECs was analyzed by western blotting relative to β-actin (n = 3). **L** The mRNA expression of IL-6, TNF-a, IL-1β, and IL-10 of si-Ctrl HUVECs and si-Piezo1 HUVECs (n = 3). Statistical significance was calculated using (**E**–**J** and **L**) Two-tailed Student’s t test and (K) two-way ANOVA, Tukey’s multiple comparisons tests. Data are expressed as mean ± SD. *P < 0.05; **P < 0.01; ***P < 0.001; ****P < 0.0001, ns, not significant

As an important part of the vascular barrier, endothelial cell dysfunction is a common event in the pathological mechanism of various diabetic cardiovascular complications, and oxidative stress is a key factor in endothelial dysfunction under hyperglycemia [31–33]. Under high glucose conditions, impaired mitochondrial function in endothelial cells leads to the accumulation of intracellular ROS. Before we examined whether Piezo1 activation could aggravate ROS production induced by HG, we initially assessed the robust responsiveness of Piezo1 channels to 2 μM Yoda1. (Additional file 2: Fig. S2C) [34]. The ROS production results showed that stimulation with Yoda1 significantly increased intracellular ROS levels in HG-incubated HUVECs (Fig. 4A, D, E). In contrast, si-Piezo1 HUVECs significantly reduced the level of ROS compared to si-Ctrl HUVECs under HG culture conditions (Fig. 5A, D, E). Correspondingly, superoxide production in HG-exposed HUVECs was also increased by treatment with Yoda1 (Fig. 4J); however, it was significantly reduced upon loss of Piezo1 expression (Fig. 5J), suggesting the involvement of superoxide in Piezo1 activation for regulation of endothelial dysfunction. Next, HUVECs were stained with the mitochondria-specific dye MitoSOX to determine the effect of Piezo1 on mitochondrial superoxide levels. As shown, Yoda1 treatment tended to aggravate HG-induced superoxide generation, as revealed by an increase in MitoSOX fluorescence intensity compared to NG and MAN induction (Fig. 4B, F). Mitochondrial depolarization was monitored by JC-1 staining. The results showed that Yoda1 stimulation of HG-treated HUVECs significantly enhanced mitochondrial aggregation indicated by red fluorescence intensity. In contrast, Piezo1 activation did not significantly alter the mitochondrial polarization of HUVECs treated with NG or NG + MAN (Fig. 4C, G). Consistent with these results, the knockdown of Piezo1 normalized the membrane potential of HG-treated HUVECs (Fig. 5B, C, F, G). These results suggested that the effect of Piezo1 activation on HG-induced endothelial injury may be related to its participation in the production of ROS and the increase in mitochondrial superoxide levels. To further evaluate the altered oxidative status of HUVECs, we examined superoxide dismutase (SOD) activity and malondialdehyde (MDA) content in culture supernatants under different culture conditions. The results showed that HG-incubated HUVECs produced higher MDA levels and showed lower SOD activity after Yoda1 stimulation, which were not observed under NG and NG + MAN culture conditions (Fig. 4H, I). After knocking down the expression of Piezo1, HUVECs in the HG group also showed lower MDA production and higher SOD activity when stimulated by Yoda1 (Fig. 5H, I), thereby indicating that Piezo1 is involved in the reduction of antioxidant capacity and increase in the oxidative capacity of endothelial cells with hyperglycemia. The secretion of the inflammatory cytokines, interleukin (IL)-6, IL-1β, and tumor necrosis factor (TNF)-β, in HUVECs treated with Yoda1 was also examined. Not surprisingly, we found that the increased secretion of inflammatory cytokines and the decreased secretion of the anti-inflammatory cytokine, IL-10 in the HG group coincided well with the presence of Yoda1, but was not significantly affected in the NG and NG + MAN groups (Fig. 4L, Additional file 2: Fig. S2A). However, the elevated levels of IL-6, IL-1β, and TNF-α caused by HG were also significantly inhibited after si-Piezo1 treatment (Fig. 5L, Additional file 2: Fig. S2B). Therefore, we suggested that Piezo1 activation may cause or accompany inflammation, aggravating endothelial dysfunction induced by HG.

### Piezo1 mediates the enhanced oxidative stress response through Ca^2+^/CaMKII and Nrf2/HO-1/NQO1 signaling pathways

**Fig. 6:**
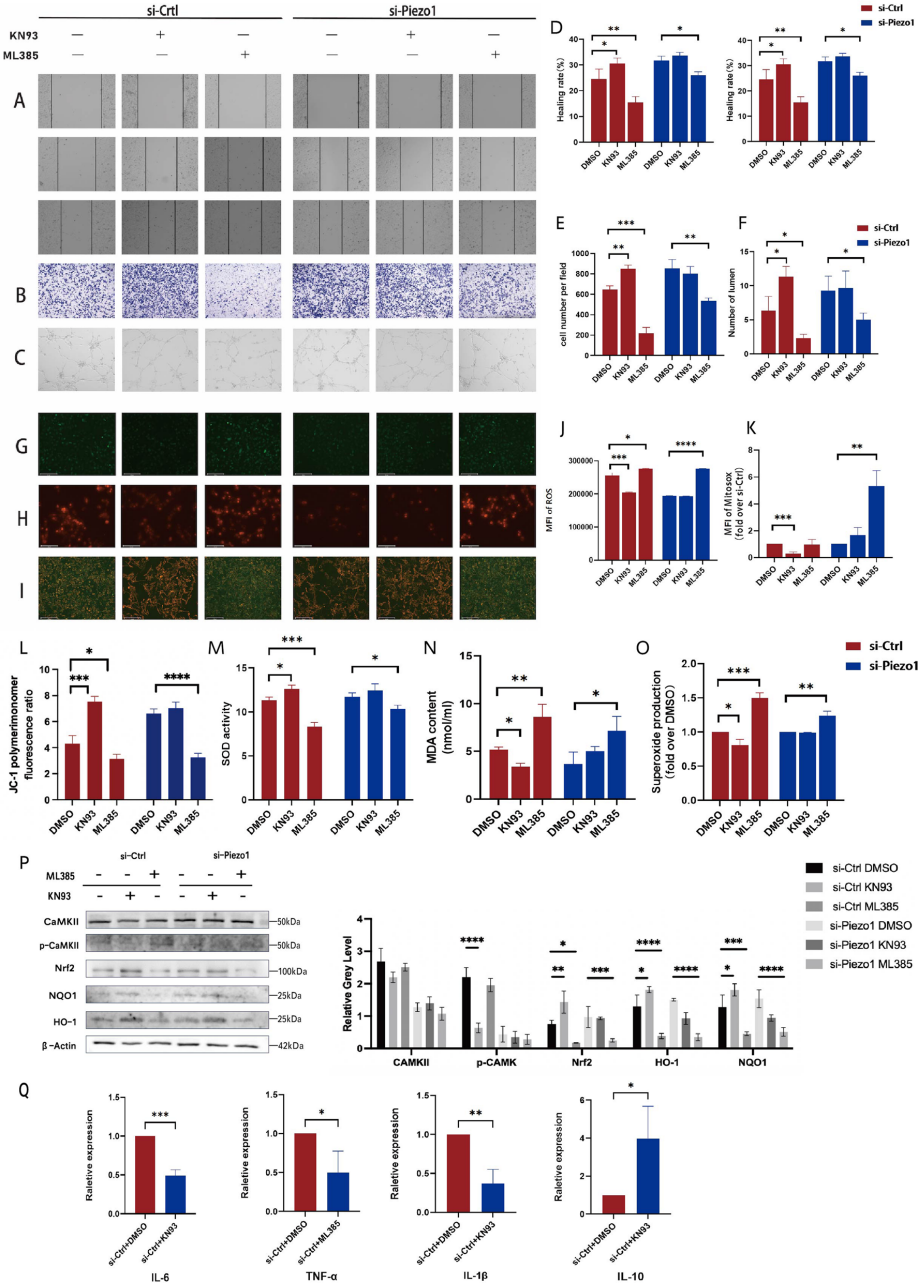
High glucose-induced HUVECs oxidative stress and dysfunction can be alleviated by KN93 or aggravated by ML385. Si-Ctrl HUVECs and si-Piezo1 HUVECs were cultured in NG condition (5.5 mM glucose) and stimulated by Yoda1 (2 μM), with or without KN93 (10 μM, 30 min) or ML385 (10 mM, 30 min) pretreatment. **A** Representative images of wound healing assay photographed at 0, 12, and 24 h of culture (100 ×). **B** Representative images of the Transwell assay photographed at 16 h of culture (200 ×). **C** Representative images of angiogenesis assay photographed at 20 h of culture (100 ×). **D** The healing rates of si-Ctrl and si-Piezo1 HUVECs at 12 and 24 h of culture (n = 3). **E** The number of cells that passed through the chamber after 16 h of culture (n = 3). **F** The number of lumens formed by si-Ctrl and si-Piezo1 HUVECs at 20 h of culture (n = 3). **G** Representative confocal images of ROS production (100 ×). **H** Representative confocal images of MitoSOX stained si-Ctrl and si-Piezo1 HUVECs (200 ×). **I** Representative confocal images of mitochondrial membrane potential detected by JC-1 fluorescence staining (100 ×). **J** ROS production of si-Ctrl and si-Piezo1 HUVECs indicated by the mean fluorescence intensity (n = 3). **K** Mitochondrial superoxide production of si-Ctrl and si-Piezo1 HUVECs measured by mitochondria-targeted probe MitoSOX (n = 3). **L** The ratio of polymer monomers to monomers indicated by JC-1 fluorescence. **M** SOD activity of si-Ctrl and si-Piezo1 HUVECs measured by ELISA (n = 3). **N** MDA content of si-Ctrl and si-Piezo1 HUVECs measured by ELISA (n = 3). **O** Relative superoxide production of si-Ctrl and si-Piezo1 HUVECs measured by ELISA (n = 3). **P** Totol-CaMKII, phosphor-CaMKII, Nrf2, NQO1, and HO-1 expression of si-Ctrl and si-Piezo1 HUVECs analyzed by western blot relative to β-actin (n = 3). **Q** The mRNA expression of IL-6, TNF-a, IL-1β, and IL-10 of si-Ctrl and si-Piezo1 HUVECs (n = 3). Statistical significances were calculated using (**P**) two-way ANOVA, (**D**–**F**, **J**–**O**) one-way ANOVA, Tukey’s multiple comparisons tests, (**Q**) Two tailed student’s t test. Data are expressed as the mean ± SD. *P < 0.05; **P < 0.01; ***P < 0.001; ****P < 0.0001, ns, not significant

Piezo1 is an ion channel characterized by a high Ca^2+^ permeability; therefore, we further examined CaMKII and its phosphorylated form (p-CaMKII) as downstream signaling molecules of Piezo1 (Figs. 4K, 5K). We found that the expression of p-CaMKII increased in HUVECs exposed to HG compared to those exposed to NG and high osmotic pressure. Moreover, Yoda1 stimulation further enhanced the expression of p-CaMKII under HG conditions, but did not significantly affect its expression in the NG and NG + MAN groups. Studies have shown that Nrf2 is a transcription factor closely related to oxidative stress that plays anti-inflammatory and antioxidative roles in diabetes [8]. Nrf2-related pathways were also enriched by gene set enrichment analysis (GSEA) of the published RNA-seq data of induced pluripotent stem cell-derived endothelial cells in diet-induced hyperglycemic obese mice (GSE54387) (Additional file 2: Fig. S2D); thus, we examined the expression of Nrf2 and its downstream molecules after Piezo1 activation. Combined treatment with HG and Yoda1 significantly inhibited the expression of Nrf2 as well as its downstream targets HO-1 and NQO1(Fig. 4K). In contrast, the knockdown of Piezo1 significantly suppressed the Yoda1-induced increase in p-CaMKII expression and enhanced Nrf2 expression, together with HO-1 and NQO1 in HG medium (Fig. 5K). However, the expression of these proteins was not strongly associated with Piezo1 activation in NG or NG + MAN groups. These data suggest that Yoda1-induced Piezo1 activation mediates the reduction in antioxidant capacity and functional impairment of HUVEC through the Ca^2+^/CaMKII and Nrf2/HO-1/NQO1 pathways. In addition, si-Ctrl HUVECs and si-Piezo1 HUVECs were pretreated with KN93, an inhibitor of CaMKII, or ML385, an inhibitor of Nrf2, before the administration with Yoda1 to explore the relationship between the above signals. We found that KN93 inhibited Yoda1-enhanced p-CaMKII expression and increased Nrf2 expression under HG conditions, whereas ML385 caused a sharp decrease in Nrf2 expression but did not affect Yoda1-induced p-CaMKII expression (Fig. 6P). In line with this, KN93 not only reduced the increase in apoptosis (Additional file 2: Fig. S2E-F), but also restored the migration (Fig. 6A, B, D, E) and tube formation (Fig. 6C, F) of si-Ctrl HUVECs treated with HG. Meanwhile, the addition of KN93 alleviated the increase in ROS production (Fig. 6G, J) and oxidation levels (Fig. 6M–O) caused by the HG and Yoda1 combined treatment, repaired the imbalance in mitochondrial membrane potential (Fig. 6H, I, K, L), and rescued the secretion of inflammatory factors (Fig. 6Q). In contrast, ML385 aggravated the apoptosis of HUVEC (Additional file 2: Fig. S2E-F), impaired the migration ability of HUVEC, as detected by Transwell and wound healing assays (Fig. 6A, B, D, E), and prevented them from forming normal tube structures in vitro (Fig. 6C, F). Moreover, ML385 significantly increased the degree of oxidative stress in HUVECs, as reflected by the accumulation of oxidative stress products and mitochondrial dysfunction (Fig. 6G–L), accompanied by increased secretion of inflammatory factors (Additional file 2: Fig. S2G). Also, inhibition of Nrf2 further impaired cell oxidation induced by HG (Fig. 6M–O). These results suggested that the antioxidant capacity of HUVEC was destroyed by Piezo1 activation under HG conditions, resulting in severe dysfunction by activation of the Ca^2+^/CaMKII pathway and inhibition of Nrf2 and its downstream molecules.

### Endothelium-specific Piezo1 deficiency alleviated murine oxidative stress in STZ-induced hyperglycemia

**Fig. 7:**
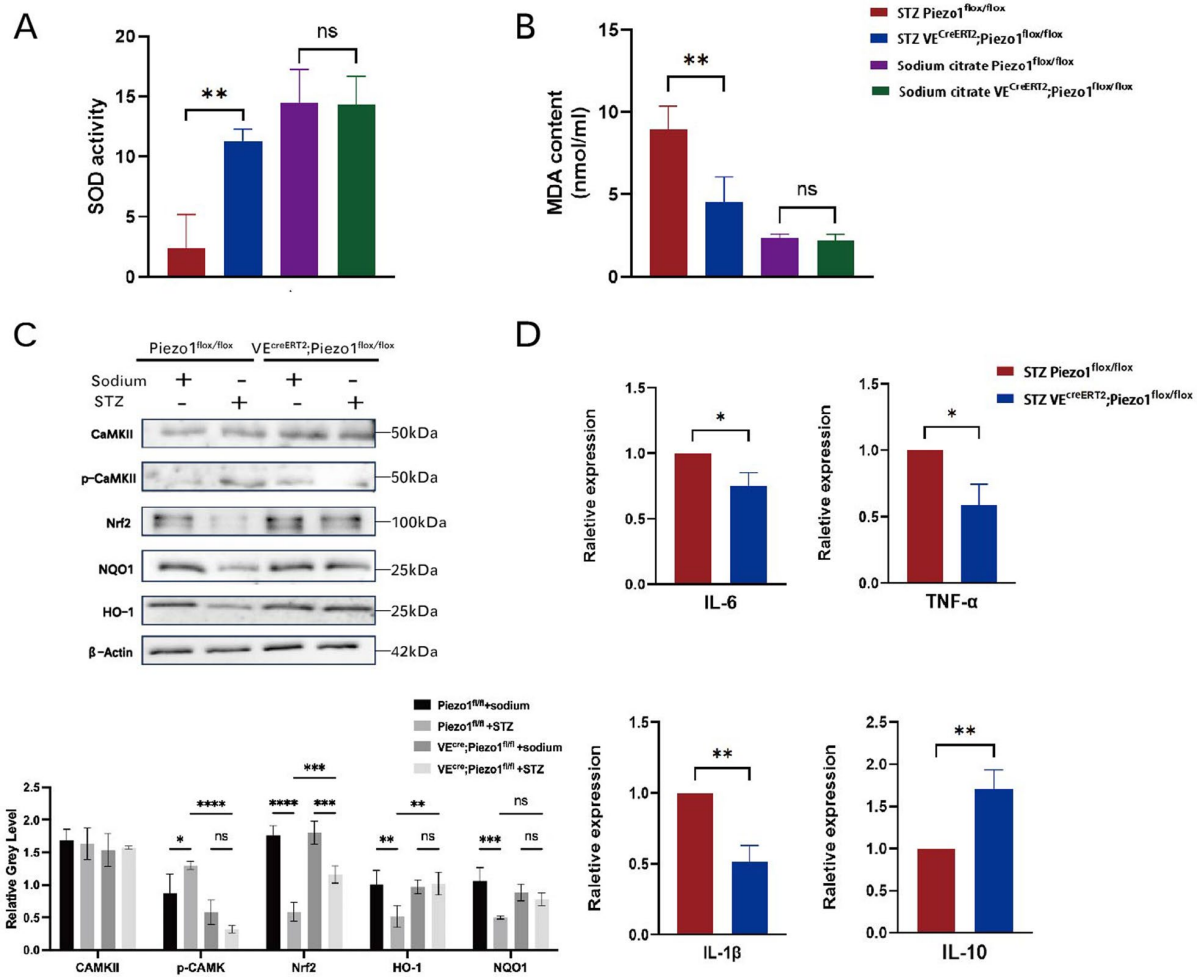
Endothelium-specific Piezo1 deficiency alleviated murine oxidative stress in STZ-induced diabetes. Piezo1^flox/flox^ and VE^creERT2^;Piezo1^flox/flox^ mice were administrated with 80cmg/kg/d STZ or sodium citrate by intraperitoneal injection after overnight fasting for three consecutive days. **A** SOD activity in mice thoracic aortic tissues (n = 3). **B** MDA content of mice thoracic aortic tissues (n = 3). **C** Totol-CaMKII, phospho-CaMKII, Nrf2, NQO1, and HO-1 expression of mice thoracic aortic tissues analyzed by western blot relative to β-actin (n = 3). **D** The mRNA expression of IL-6, TNF-a, IL-1β, and IL-10 of mice thoracic aortic tissues (n = 3). Statistical significances were calculated using (**A**–**C**) two-way ANOVA, Tukey’s multiple comparisons tests, (D)two tailed student’s t test. Data are expressed as the mean ± SD. *P < 0.05; **P < 0.01; ****P < 0.0001, ns, not significant

Both, the increase in blood viscosity caused by hyperglycemia and the deformation caused by alterations in cell osmotic pressure can lead to the changes in mechanical force in the lumen. To directly verify whether Piezo1 deficiency affects the level of HG-induced endothelial oxidative stress, thoracic aortas were isolated from Piezo1^flox/flox^ and VE^CreERT2^;Piezo1^flox/flox^ mice 6 months after STZ administration. We found a significant decline in SOD activity and an increase in MDA content in Piezo1^flox/flox^ mice compared to healthy controls and VE^creERT2^;Piezo1^flox/flox^ mice (Fig. 7A, B). Consistent with that showed in si-Piezo1 HUVECs, endothelial Piezo1 deficient mice also showed reduced mRNA levels of IL-6, IL-1β, and TNF-α, accompanied by an increase in IL-10 (Fig. 7D). We also examined the expression of Ca^2+^/CaMKII and Nrf2/HO-1/NQO1 pathway molecules at the protein level. VE^creERT2^;Piezo1^flox/flox^ mice showed decreased p-CaMKII expression compared with Piezo1^flox/flox^ mice and increased expression of Nrf2 and its downstream molecules HO-1 and NQO1 (Fig. 7C). These results indicate that endothelial Piezo1 deficient mice experienced a mild oxidative stress response in STZ-induced hyperglycemia, confirming that Piezo1 is also involved in HG-induced oxidative stress injury in vivo.

## Discussion

Diabetes causes vascular lesions and abnormal blood flow, which can be detected by endothelial cells located in the innermost part of the vessel wall. In this study, we found that conditionally endothelial Piezo1-deficient transgenic mice ameliorated STZ-induced hyperglycemia by limiting oxidative stress and impairing endothelial barriers. Meanwhile, Piezo1 knockdown in vitro confirmed that Piezo1 activation aggravated high glucose-induced oxidative stress damage in HUVECs cell line and resulted in severe dysfunction by activation of the Ca^2+^/CaMKII pathway and inhibition of Nrf2 and its downstream molecules. Combining the results from in vitro and in vivo experiments, our research indicated Piezo1 activation under hyperglycemia and its adverse role in high glucose-induced oxidative stress injury, offering new insights into endothelial dysfunction in hyperglycemia.

Endothelial cells are more susceptible to hyperglycemia-induced cellular damage than other cell types, including fatty acid oxidation, nitric oxide decrease, oxidative stress, inflammatory activation, and impaired barrier function [4].To date, widely accepted hypotheses regarding the contribution of hyperglycemia to diabetic complications include heightened polyol pathway flux, activation of protein kinase C (PKC) isoforms, enhanced creation of advanced glycation end products (AGEs), and augmented hexosamine pathway flux [35]. In this study, we attempted to determine the role of endothelial cells as sensors of mechanical stress under a hyperglycemia condition. Endothelial cells are the outermost layer covering the lumen of blood vessels and can sense the mechanical force of blood flow and protect cell surface receptors from overactivation [36]. The presence of pulsatile blood flow within the circulatory system, accompanied by the movement of formed elements, particularly red blood cells, induces a perpetually fluctuating force profile on the endothelial cell layer [37]. Diabetes considerably increases the viscoelastic and thixotropic behavior of blood thixotropy, which contributes to the nonuniformity of blood flow patterns and hemodynamic forces in the vascular system [1]. Therefore, it is reasonable to speculate that endothelial cell sensitivity to mechanical force may play a role in high glucose-induced endothelial cell injury and lead to functional alterations.

The mechanisms by which endothelial cells sense mechanical forces are complex. Piezo1 is a widely studied non-selective mechanosensitive ion channel that plays a key role in vascular diseases. Multiple studies have shown that Piezo1 has significant effects on vascular development and participates in the physiology and disease states of vascular mechanobiology and related clinical diseases, such as atherosclerosis and hypertension [38].

Moreover, several studies have reported the involvement of Piezo1 in diabetes and high glucose conditions. Research has indicated that deregulation of Piezo1 has been observed in several lineages of individuals diagnosed with type 2 diabetic mellitus (T2DM), which can promote Piezo1 transcription in mature blood cells and specific hematopoietic stem cell clones with high Piezo1 expression for cloning [26]. Piezo1 is also known to be involved in glucose tolerance maintenance, glucose-induced insulin secretion, and islet β-cell electrophysiological activity [39]. Meanwhile, it has been reported that Piezo1 inhibition ameliorates acute hyper glucose-induced brain microglial injury [40]. Additionally, the inhibition of Piezo1 has been shown to protect against hyperglycemia-induced retinal light conduction damage in mice [41]. Similarly, our study revealed that Piezo1 activation exacerbates endothelial cell dysfunction in a high-glucose environment. Current evidence suggests that a key role of Piezo1 in the endothelium is to regulate the release of NO [42]. Flow-induced NO production plays an important role in the adaptation of the vessel diameter to hemodynamic forces and the corresponding control of vascular tone and blood pressure, which are associated with the activation of the inflammatory phenotype of endothelial cells or the formation of atherosclerosis [43]. Recently, a study has revealed the activation of Piezo1 and its impact on phosphorylated Ca^2+^/CAMKII and phosphorylated eNOS in HUVECs, and found that the acute effects of Piezo1 activation inhibit vasodilation in small resistance arteries under hyperglycemia [42]. Correspondingly, we detected the expression of eNOS in the endothelial cells of the aorta from hyperglycemic mice with endothelial-specific knockout of Piezo1, confirming that the knockout of Piezo1 alleviates the potential endothelial cell dysfunction induced by high glucose. However, our results suggested a novel mechanism by which Piezo1 regulates endothelial function by participation in oxidative stress.

Hyperglycemia-induced oxidative stress and DNA damage play important roles in endothelial cell impairment [44] and development of diabetes complications [45]. A substantial quantity of glucose in the blood enters the tricarboxylic acid cycle, thereby additional electron donors (NADH and FADH2) are pushed into the electron transport chain, which increases mitochondrial transmembrane voltage and forms superoxide-dominant ROS [46]. Metabolic dysregulation in diabetes leads to an excessive generation of mitochondrial superoxide inside the endothelial cells of blood vessels [45], which damages endothelial cells by activation of poly(ADP-ribose) to inhibit GAPDH [47]. Many studies have shown that Ca^2+^ and ROS signaling pathways overlap and influence each other [48]. Ca^2+^ plays a crucial role in modulating the membrane potential, governing mitochondrial adenosine triphosphate (ATP) synthesis, and regulating protein function, including calcineurin (CaN) and calmodulin (CaM) [49, 50]. Evidence suggests that Ca^2+^ influx causes short-lived and highly reactive ROS production in cell [51]. This interaction has significant implications for the development and progression of cardiovascular, neurodegenerative, and cancerous diseases [52]. Consistent with these results, our study also demonstrated the presence of ROS production in hyperglycemia-damaged endothelial cells, which is closely related to Ca^2+^ influx caused by Piezo1 activation. However, Piezo1-dependent Ca^2+^ influx modulate ROS generation and homeostasis, which subsequently enhances the release of Ca^2+^ to produce a positive feedback balance between Piezo1 distribution and expression and ROS production to maintain normal physiological conditions [21]. Thus, whether the imbalance between Ca^2+^ influx and ROS production under HG conditions promotes each other and aggravates vascular endothelial injury remains to be studied.

Mechanistically, elevated Ca^2+^ in mouse embryonic cells induces ROS production, which upregulates Nrf2 and its target genes [53]. Human amniotic mesenchymal stem cell senescence can be delayed by targeting the CaM/CaMKII signaling pathway and inhibiting Nrf2-mediated antioxidant function [54]. Consistently, the Nrf2-related pathway was also enriched by GSEA of published RNA-seq data of induced pluripotent stem cell-derived endothelial cells in diet-induced hyperglycemic obese mice (GSE54387). Nrf2 has been shown to be involved in protection of cells from calcium-induced oxidative stress in degenerative nerves [55] and stressed lens cells [56], and is also associated with the regulation of mitochondrial homeostasis [57] and Ca^2+^-related epithelial–mesenchymal transition in hepatocellular carcinoma cells [58]. Decreased Nrf2 levels have been observed in models of diabetic nephropathy and osteoporosis. Treatment with fenofibrate or osteoporosis can upregulate Nrf2 to inhibit diabetes-associated ferroptosis [59, 60]. Moreover, studies have shown that specific inhibition of histone deacetylase 3 (HDAC3) can significantly improve the blood–brain barrier permeability and downregulate connexin expression in diabetic mice by targeting the Nrf2 signaling pathway and its downstream molecules, thus becoming a new target for the treatment of blood–brain barrier damage in T2DM [61]. In addition, evidence shows that hyperglucose-induced endothelial cells downregulate Nrf2 expression and have a metabolic memory that is not erased when switched to hypoglycemia, which leads to perivascular fibrosis and cardiac dysfunction [62]. Coincidentally, decreased Nrf2 expression in diabetic endothelial cells was found in our study, accompanied by downregulation of the downstream molecules HO-1 and NQO1, indicating dysregulation of the antioxidant capacity of endothelial cells under HG conditions. Therefore, our study proposes a novel mechanism for the oxidative stress response triggered by Ca^2+^ influx due to Piezo1 activation in endothelial cells under high glucose conditions, which provides an important clue for the alleviation of high glucose-induced endothelial injury and hyperglycemic vascular dysfunction.

## Conclusion

In summary, our study indicated that Piezo1 activation aggravates endothelial oxidative stress injury and the inflammatory response in a high-glucose environment, suggesting a novel way for Piezo1 to regulate endothelial function by participation in oxidative stress during hyperglycemia, which proposed a novel approach for alleviating endothelial cell damage induced by high glucose.

### Supplementary Information

**Additional file 1: Fig. S1**. The impact of Piezo1 activation on the proliferation of HUVECs cell line.

**Additional file 2: Fig. S2**. The impact of Piezo1 activation on the generation of inflammatory factors and Nrf2/HO-1/NQO1 signaling pathways.

** Additional file 3: Table S1**. PCR primer sequences and siRNA sequences.

## Data Availability

The datasets used and/or analyzed during the current study are available from the corresponding author on reasonable request.

## Abbreviations

ROS: Reactive oxygen species
NO: Nitric oxide
STZ: Streptozotocin
CaMKII: Calmodulin-dependent protein kinase II
HUVECs: Human umbilical vein endothelial cells
SOD: Superoxide dismutase
MDA: Malondialdehyde
TUNEL: TdT-mediated dUTP Nick-End Labeling
eNOS: Endothelial nitric oxide synthase
NG: Normal glucose
HG: High glucose
MAN: Mannitol
Nrf2: NF-E2-related factor 2
HO-1: Heme oxygenase 1
NQO1: NAD(P)H: quinone oxidoreductase 1
T2DM: Type 2 diabetic mellitus
RT: Room temperature
