# Supplementary figures and images for: Ion channel Piezo1 activation aggravates the endothelial dysfunction under a high glucose environment
Source: Cardiovasc Diabetol. 2024 May 3;23:150. doi: 10.1186/s12933-024-02238-7 (PMC11067304; doi:10.1186/s12933-024-02238-7)

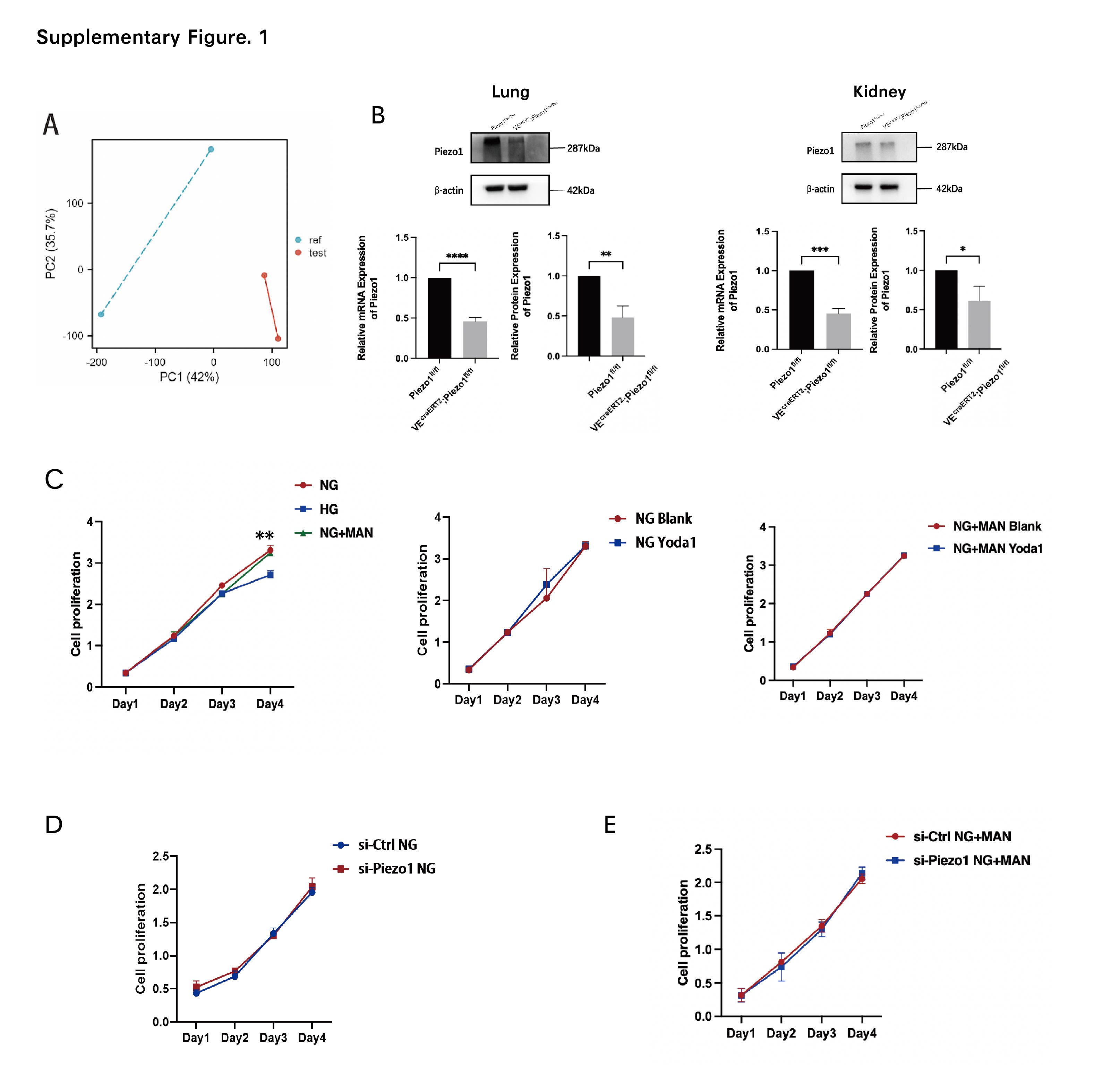

Supplement: Supplementary file 1 — Additional file 1: Fig. S1. The impact of Piezo1 activation on the proliferation of HUVECs cell line. [file 12933_2024_2238_MOESM1_ESM.jpg]

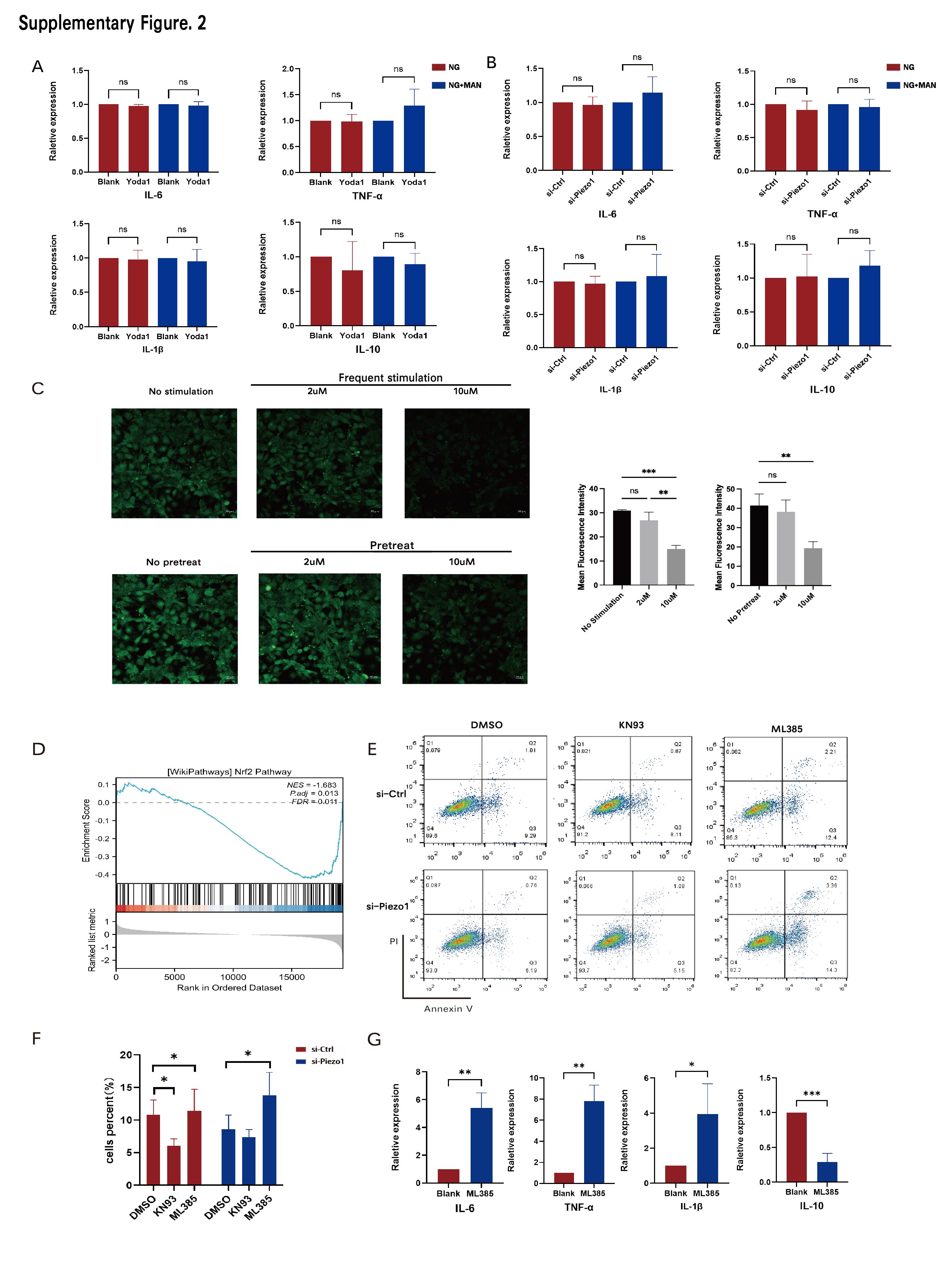

Supplement: Supplementary file 2 — Additional file 2: Fig. S2. The impact of Piezo1 activation on the generation of inflammatory factors and Nrf2/HO-1/NQO1 signaling pathways. [file 12933_2024_2238_MOESM2_ESM.jpg]
