# Supplementary material for: Ion channel Piezo1 activation aggravates the endothelial dysfunction under a high glucose environment
Source: Cardiovasc Diabetol. 2024 May 3;23:150. doi: 10.1186/s12933-024-02238-7 (PMC11067304; doi:10.1186/s12933-024-02238-7)
Supplement: Supplementary file 3 — Additional file 3: Table S1. PCR primer sequences and siRNA sequences. [file 12933_2024_2238_MOESM3_ESM.docx]

**Supplementary Table1：PCR primer sequences and siRNA sequences.**

| **Target Gene** | **Forward Primer** | **Reverse Primer** |
| --- | --- | --- |
| Mus-IL-1β | GTGTCTTTCCCGTGGACCTT | AATGGGAACGTCACACACCA |
| Mus-IL-6 | CTTCTTGGGACTGATGCTGGT | CTCTGTGAAGTCTCCTCTCCG |
| Mus-IL-10 | GCTCTTGCACTACCAAAGCC | CTGCTGATCCTCATGCCAGT |
| Mus-TNF-α | CGGGCAGGTCTACTTTGGAG | ACCCTGAGCCATAATCCCCT |
| Homo-IL-1β | CAACAAGTGGTGTTCTCCATGTC | ACACGCAGGACAGGTACAGA |
| Homo-IL-6 | CAATGAGGAGACTTGCCTGGT | GCAGGAACTGGATCAGGACT |
| Homo-IL-10 | CCAGACATCAAGGCGCATGT | GATGCCTTTCTCTTGGAGCTTATT |
| Homo-TNF-α | GAGGCCAAGCCCTGGTATG | CGGGCCGATTGATCTCAGC |
| Mus-Piezo1 | GTTACCCCCTGGGAACATCT | TTCAGGAGAGAGGTGGCTGT |
| Homo-Piezo1 | GACGCCTCACGAGGAAAG | GTCGTCATCATCGTCATCGT |
| Mus-GAPDH | AGGTCGGTGTGAACGGATTTG | TGTAGACCATGTAGTTGAGGTCA |
| Homo-GAPDH | GAAGGTGAAGGTCGGAGTC | GAAGATGGTGATGGGATTTC |
| si-Piezo1 | GGUCCUACCUUGACAUGCUTT | AGCAUGUCAAGGUAGGACCTT |
| si-Ctrl | UUCUCCGAACGUACGUTT | ACGUGACACGUUCGGAGAATT |
